# Ocular Surface Temperature in Age-Related Macular Degeneration

**DOI:** 10.1155/2014/281010

**Published:** 2014-11-11

**Authors:** Andrea Sodi, Sara Matteoli, Giovanni Giacomelli, Lucia Finocchio, Andrea Corvi, Ugo Menchini

**Affiliations:** ^1^Department of Translational Surgery and Medicine, Eye Clinic, University of Florence, Largo Brambilla 3, 50134 Florence, Italy; ^2^Laboratory of Ocular Thermography, Department of Industrial Engineering, University of Florence, Florence, Italy

## Abstract

*Background*. The aim of this study is to investigate the ocular thermographic profiles in age-related macular degeneration (AMD) eyes and age-matched controls to detect possible hemodynamic abnormalities, which could be involved in the pathogenesis of the disease. *Methods*. 32 eyes with early AMD, 37 eyes with atrophic AMD, 30 eyes affected by untreated neovascular AMD, and 43 eyes with fibrotic AMD were included. The control group consisted of 44 healthy eyes. Exclusion criteria were represented by any other ocular diseases other than AMD, tear film abnormalities, systemic cardiovascular abnormalities, diabetes mellitus, and a body temperature higher than 37.5°C. A total of 186 eyes without pupil dilation were investigated by infrared thermography (FLIR A320). The ocular surface temperature (OST) of three ocular points was calculated by means of an image processing technique from the infrared images. Two-sample *t*-test and one-way analysis of variance (ANOVA) test were used for statistical analyses. *Results*. ANOVA analyses showed no significant differences among AMD groups (*P* value >0.272). OST in AMD patients was significantly lower than in controls (*P* > 0.05). *Conclusions*. Considering the possible relationship between ocular blood flow and OST, these findings might support the central role of ischemia in the pathogenesis of AMD.

## 1. Introduction

Age-related macular degeneration (AMD) represents the primary cause of visual deterioration and legal blindness in patients over 60 years old [1] and the third leading cause worldwide [2]. It is a complex and multifactorial disease due to degenerative changes of the choroid and choriocapillaris, the retinal pigmented epithelium (RPE), Bruch's membrane, and photoreceptors [3–5] but the histopathological mechanisms are not completely clarified.

The International Classification and Grading System for age-related maculopathy and age-related macular degeneration recognizes an early stage of the disease (age-related maculopathy or ARM) and a late stage (AMD) divided in dry AMD (or geographic atrophy) and wet AMD (or neovascular AMD) [6].

Despite intensive research, the pathogenic mechanisms of AMD are poorly understood. The role of genetics has been widely confirmed [7–11] as suggested by the recurrence of the disease in some families [12] and the identification of several loci associated with a higher risk of AMD [13–19]. Oxidative damage [20] and an abnormal inflammatory response [7, 8, 21–29] have been also implicated in AMD development.

Furthermore, some authors strongly support the hypothesis of an ischemic etiology of the disease [30–34] related to choroidal and retinal blood flow abnormalities [31–40].

Fluorescein angiography and indocyanine green angiography [39, 40], laser Doppler flowmeter [31–33, 39, 41], ocular blood flow tonometry [42], and digitised ultrasound [43] have allowed evaluating ocular blood flow alterations in patients with AMD, sustaining the idea that a vascular ischemic mechanism plays a central role in the pathogenesis of the disease.

In the present study polypoidal choroidal vasculopathy (PCV) was excluded as high flow values in this relatively rare subtype of macular degeneration might have biased our estimates of choroidal blood flow in AMD [44–46].

Infrared thermography has been used to measure ocular surface temperature (OST) which is believed to represent an indirect marker of ocular hemodynamics: a possible correlation between OST and ocular blood flow has been suggested by several previous studies [47–55].

At present no information about OST in AMD is available.

The aim of this study is to investigate the ocular thermographic profiles in AMD eyes to detect possible hemodynamic abnormalities, which could be involved in the pathogenesis of the disease.

## 2. Materials and Methods

One hundred and eighteen patients (34 M/84 F, 79 ± 2 years) affected by AMD and 44 healthy subjects (21 F/23 M, 72 ± 7 years) were enrolled and recruited at the Eye Clinic, Department of Surgery and Translational Medicine, University of Florence, Italy. The study followed the tenets of the Declaration of Helsinki and was approved by the Institutional Review Board at the University of Florence. Informed consent was obtained from each patient after explanation of the purpose and description of the procedures of the study. The presence of other ocular or systemic pathologies was carefully investigated. Specifically, exclusion criteria were glaucomatous optic neuropathy, high myopia, retinal angiomatous proliferation (RAP), polypoidal choroidal vasculopathy (PCV), and other retinal and choroidal diseases except for typical AMD, corneal or tear film abnormalities, and diabetes mellitus and a body temperature higher than 37.5°C.

All the subjects included in the study underwent a comprehensive clinical evaluation including best corrected visual acuity measurement, anterior segment evaluation, tonometry, and biomicroscopy of the posterior pole.

Optical coherence tomography (OCT) scan (Topcon 3D OCT-1000, Topcon Medical Systems Inc, Oakland, NJ, USA) and/or fluorescein angiography (FA) was performed when active choroidal neovascularization (CNV) was diagnosed or suspected.

Three observers (AS, GG, and LF) evaluated independently all eyes. The same observers classified the 142 selected AMD eyes into four subsets depending on the form of AMD diagnosed at the time of the thermographic acquisition. Specifically, we divided the AMD eyes into the following groups: twenty-nine patients (32 eyes) with ARM (20 F/9 M, 77 ± 7 years), 27 patients (37 eyes) with atrophic AMD (19 F/7 M, 82 ± 6 years), 29 patients (30 eyes) affected by neovascular AMD (18 F/11 M, 77 ± 7 years), and 33 patients (43 eyes) with fibrotic AMD (27 F/6 M, 79 ± 7 years) were included in the study.

None of the neovascular AMD patients had ever been treated with photodynamic therapy or intravitreal antiangiogenic drugs prior to thermographic examination. Specifically, these patients underwent infrared thermography before the first planned intravitreal injection on the same day.

Fibrotic AMD group consisted of eyes with previous wet AMD which evolved to a fibrous macular scar with or without treatment that did not present any sign of vascular activity (angiographic leakage or OCT fluid) at the time of the thermographic measurement.

The control group consisted of 44 age-matched subjects with healthy eyes who underwent routine clinical examination. One eye was randomly chosen for thermographic evaluation.

A total of 186 eyes (142 AMD and 44 controls) were investigated by infrared thermography.

The pupils were not dilated in order to avoid possible influence of pupil diameter on the thermographic profile.

The thermocamera used was the FLIR A320 (FLIR System, USA) with an image resolution of 320 × 240 pixels and image frequency of 30 Hz. The detectors time constant was 12 ms with accuracy ±2°C/±2% and sensibility of 0.05°C at +30°C. OST measurements were carried out by only one examiner in order to avoid interexaminer variation, in a room without windows, illuminated with neon lights. Both temperature and humidity were constantly monitored and maintained to an average of 20.8 ± 2.7°C and 42 ± 9% by using an air conditioning system.

The same procedure was applied for each thermographic acquisition. Subjects remained in the test-room for 20 minutes, so that their own body temperature could adapt to the climatic condition of test-room. Then, subject's chin was positioned on an ophthalmic chinrest in front of the thermocamera, whose lens was positioned at 300 mm. The subject was asked to keep both eyes closed for 10 s before starting the measurement and to keep both eyes widely open during the thermographic acquisition (7 s at 30 Hz), so that just one recording was sufficient for evaluating both eyes. Three recordings were taken for each subject.

**Figure 1:**
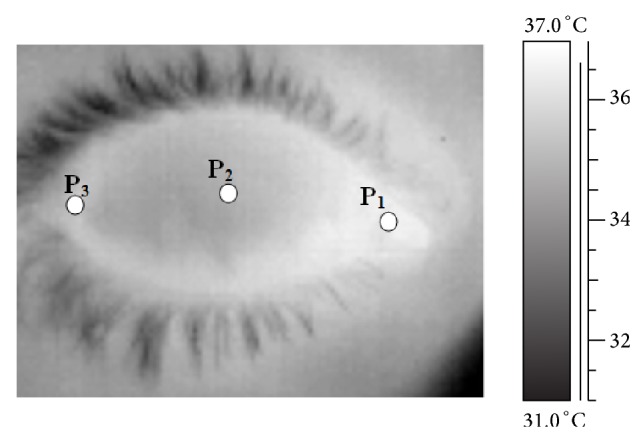
Infrared image of an eye. 1 is nasal cantus, 2 is corneal centre, and 3 is temporal cantus.

For each thermographic acquisition only the first frame corresponding to the eye opening was selected for further analysis, in order to avoid the influence of the tear-film evaporation. A Matlab code (R2009b, Mathworks, USA) was used to calculate, from the selected frames, the temperatures of three anatomical points corresponding to the principal anatomical areas of the anterior eye: corneal centre (P_2_) and temporal and nasal* canti* (P_3_ and P_1_), as shown in Figure 1.

Analysis of variance (*one-way ANOVA*, Stata 12.1 software, StataCorp, USA) was applied in order to assess the measurements repeatability as well as the difference among the temperatures of the three points selected for all groups investigated. The same statistical analysis was also used to assess whether there was a statistically significant difference in the OST among AMD groups. Furthermore,* unpaired t-test* (Stata 12.1 software, StataCorp, USA) was carried out in order to compare the entire AMD population as well as each AMD subgroup with controls. The differences were considered statistically significant when *P* value was less or equal to 0.05.

## 3. Results

For each eye the average temperature of the three recordings was considered, as* ANOVA* analyses showed no significant differences among the three recordings.

A characteristic thermographic profile characterized by higher temperatures at the extremities (P_1_ and P_3_) and a lower temperature in the corneal centre (P_2_) was found in all subjects.

*ANOVA* tests showed that there was a statistically significant difference among the temperatures of the three points for both AMD groups (*P* value < 0.0001) and healthy controls (*P* value < 0.0001).

**Figure 2:**
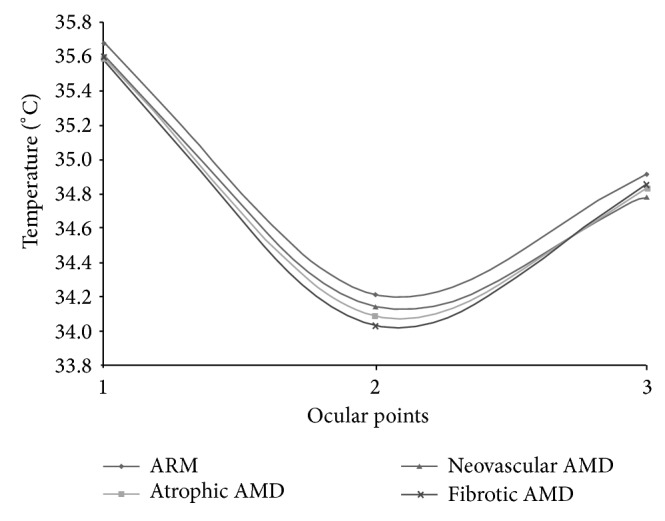
Average OST profiles of AMD patients and controls.

The average results for the four AMD subsets of patients are summarized in Table 1.* ANOVA* tests showed no significant difference among AMD groups (*P* value > 0.272), as shown in Figure 2.

**Figure 3:**
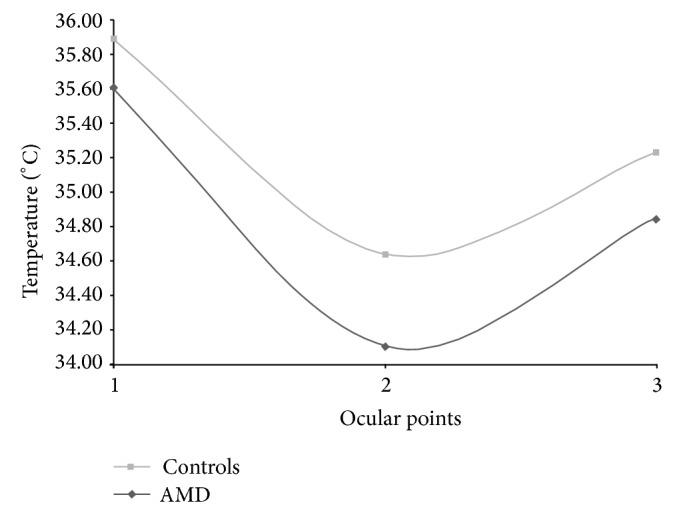
Comparison among the average OST profiles of all AMD groups.

*Unpaired t* *-test* showed a significant difference between the total AMD population and controls in all points (*P* value < 0.009), as shown in Table 2 and Figure 3.

When statistically comparing each AMD group with controls, a significant difference was found in all points (*P* value < 0.05), as shown in Table 3.

## 4. Discussion

Infrared thermography allows ocular hemodynamics evaluation by measuring the heat radiated from the eye surface. Previous studies showed OST abnormalities in retinal vascular disorders, such as arterial occlusive disease [47], central vein occlusion [52], diabetic retinopathy [53], glaucoma [54, 55], bacterial corneal ulcers [56], and dry eye syndrome [57]. In the present study we evaluated OST in patients affected by different forms of AMD.

Both AMD patients and healthy controls included in this study showed a common thermography profile with a lower temperature in the central cornea (point P_2_) and a higher temperature at the extremities of the profile, in the nasal and temporal scleroconjunctival areas (P_1_ and P_3_). This result can be explained by considering that the center of the cornea is nonvascularised and more prone to tear evaporation, while the extremities are located in areas with a relevant blood supply and less influenced by the tear evaporation.

In our study the OST of AMD patients is significantly lower than that of healthy subjects in the three chosen ocular points. As OST is indirectly associated with blood perfusion, its reduction may suggest a decrease in ocular blood flow. This result strengthens the central role of ischemia in the pathogenesis of AMD, in agreement with the hypothesis that impairment in choroidal circulation may represent a primary pathogenic mechanism leading to RPE senescence and AMD [58, 59]. However, an OST reduction, indirectly suggesting a blood flow decrease, does not support the role of inflammation in the pathogenesis of the disease. In fact, inflammation is usually associated with an increase in blood perfusion that should lead to OST increase. Of course our data does not exclude a role of inflammation in AMD onset and progression because very limited inflammatory processes may determine a relevant functional impact, in spite of the poor influence on ocular hemodynamics and OST.

In the present study early or advanced and atrophic or neovascular AMD do not show significant differences in the OST. All AMD subgroups show a reduced surface temperature value suggesting that hemodynamic abnormalities may represent a common pathogenic pathway for the different forms and stages of the disease. The physiopathological affinity between atrophic and neovascular AMD is in agreement with that encountered in clinical practice where some patients may show a dry AMD in one eye and an exudative AMD in the other. Similarly, some patients may start with an atrophic AMD later complicated by a CNV.

The lack of significant OST differences among the four AMD subgroups and particularly between ARM and the more advanced stages of the disease suggests that OST measurements cannot be used for an early diagnosis of CNV or for monitoring the disease progression. Probably the evaluation of the thermographic profile may be a reliable tool to appreciate large vascular changes (like the ischemic alterations involving the whole choroid), but OST measurement does not have enough sensitivity to detect very small (even if clinically very relevant) vascular abnormalities. We can speculate that thermography could be used to detect possible choroidal atrophic changes consequent to photodynamic therapy or to repeated antivascular endothelial growth factor (anti-VEGF) intravitreal injection.

Our study shows some limitations. OST evaluation is only an indirect method for ocular blood supply assessment and can be influenced by many factors (drugs assumption, vascular diseases other than diabetes, smoking history, and lifestyles). Moreover, the evaluation of OST distribution in the cornea and in the contiguous scleroconjunctival areas with our present technology provides very poor topographic information about the location of possible intraocular vascular abnormalities. These limitations are innate in the present procedure of OST evaluation and could be possibly overcome by means of technological refinements.

It would be interesting to evaluate the influence of lesion size and level of activity, as well as CNV subtypes (classic, occult, and mixed) on the OST in further investigations.

## 5. Conclusions

Infrared thermography may be a helpful, noninvasive, and not time-consuming method to be used in the evaluation of patients with AMD. It could provide interesting information about the physiopathology of the disease although at present it does not seem suitable for the management of AMD patients in a clinical setting.

## Figures and Tables

**Table 1:** Ocular surface temperature of all three points expressed as means ± one standard deviation for AMD groups and controls.

Groups	*T*1 [°C]	*T*2 [°C]	*T*3 [°C]
ARM	35.68 ± 0.42	34.21 ± 0.53	34.91 ± 0.47
Atrophic AMD	35.59 ± 0.44	34.08 ± 0.47	34.83 ± 0.54
Neovascular AMD	35.60 ± 0.61	34.14 ± 0.75	34.79 ± 0.69
Fibrotic AMD	35.58 ± 0.31	34.03 ± 0.42	34.85 ± 0.42
Controls	35.89 ± 0.52	34.64 ± 0.84	35.23 ± 0.60

**Table 2:** Ocular surface temperature of all three points expressed as means ± one standard deviation for all AMD patients and controls.

	*T*1 [°C]	*T*2 [°C]	*T*3 [°C]
AMD	35.61 ± 0.44	34.11 ± 0.54	34.85 ± 0.52
Controls	35.89 ± 0.52	34.64 ± 0.84	35.23 ± 0.60
*P* value	0.009	0.001	0.001

**Table 3:** *P* values calculated from unpaired  t-test carried out between controls and each AMD subgroup.

	Controls		
	*T*1 [°C]	*T*2 [°C]	*T*3 [°C]
ARM	0.052	0.008	0.011
Atrophic AMD	0.006	<0.0001	0.003
Neovascular AMD	0.036	0.009	0.006
Fibrotic AMD	0.001	<0.0001	0.001
